# Genome-Wide Methylation Patterns in Primary Uveal Melanoma: Development of MethylSig-UM, an Epigenomic Prognostic Signature to Improve Patient Stratification

**DOI:** 10.3390/cancers16152650

**Published:** 2024-07-25

**Authors:** Emilie Lalonde, Dong Li, Kathryn Ewens, Carol L. Shields, Arupa Ganguly

**Affiliations:** 1 Department of Genetics, Perelman School of Medicine, University of Pennsylvania, Philadelphia, PA 19104, USA; 2 Department of Pathology and Laboratory Medicine, Schulich School Medicine & Dentistry, Western University, London, ON N6A 5C1, Canada; 3 Oncology Services, Wills Eye Hospital, Thomas Jefferson University, Philadelphia, PA 19144, USA

**Keywords:** uveal melanoma, methylation, prognostic signature, epigenetic biomarker, metastasis prediction, bioinformatics

## Abstract

**Simple Summary:**

Methylation changes are epigenetic, or noncoding changes to DNA. Methylation changes in cancers are increasingly being used to identify biomarkers, novel biology, and therapeutic targets in cancer. We present a new cohort of DNA methylation in primary uveal melanoma with long-term follow-up including metastatic outcome. We identified novel differentially methylated regions and epigenomic subtypes associated with metastatic risk. To make a clinically useful biomarker, we developed a methylation signature, MethylSig-UM, that can be used at diagnosis of primary uveal melanoma to identify patients at high risk of metastasis. MethylSig-UM-positive tumors are also scored as high-risk by other established biomarkers. These positive tumors have increased expression of immune-modulating genes, which can explain the poor response of uveal melanoma to immunotherapies. Methylation profiling, therefore, represents a promising new avenue for patient prognostication in primary uveal melanoma.

**Abstract:**

Despite studies highlighting the prognostic utility of DNA methylation in primary uveal melanoma (pUM), it has not been translated into a clinically useful tool. We sought to define a methylation signature to identify newly diagnosed individuals at high risk for developing metastasis. Methylation profiling was performed on 41 patients with pUM with stage T2–T4 and at least three years of follow-up using the Illumina Infinium HumanMethylation450K BeadChip (N = 24) and the EPIC BeadChip (N = 17). Findings were validated in the TCGA cohort with known metastatic outcome (N = 69). Differentially methylated probes were identified in patients who developed metastasis. Unsupervised consensus clustering revealed three epigenomic subtypes associated with metastasis. To identify a prognostic signature, recursive feature elimination and random forest models were utilized within repeated cross-validation iterations. The 250 most commonly selected probes comprised the final signature, named MethylSig-UM. MethylSig-UM could distinguish individuals with pUM at diagnosis who develop future metastasis with an area under the curve of ~81% in the independent validation cohort, and remained significant in Cox proportional hazard models when combined with clinical features and established genomic biomarkers. Altered expression of immune-modulating genes were detected in MethylSig-UM positive tumors, providing clues for pUM resistance to immunotherapy. The MethylSig-UM model is available to enable additional validation in larger cohort sizes including T1 tumors.

## 1. Introduction

Uveal melanoma (UM) is a rare eye cancer that affects 5.1 per million people in the United States [1]. The risk of developing UM is greater in males [2] and increases with age; the median age at diagnosis is 59 years [3]. Approximately 50% of individuals diagnosed with UM develop metastasis within ten years, primarily to the liver (67%), but also to lungs and bones [4,5]. Once metastasis occurs, the prognosis becomes poor. In addition to older age and male gender, several clinical and tumor characteristics help predict the likelihood of metastasis, including changes in the copy number status of chromosome 3, 6, 8, and 1p, types of tumor cells (spindle, epithelioid or mixed), and features such as mitotic activity, cell density, and the presence of infiltrating lymphocytes [2,5,6,7,8,9,10].

Currently, most models that predicts risk of metastasis in UM rely on of a combination of high-risk factors, including large tumor height and diameter, monosomy of chromosome 3, gain of chromosome 8q, and loss of chromosome 8p, 6q, or 1p [11,12], as well as somatic or germline variants in BAP1 [13,14]. In addition, somatic variants in SF3B1 and EIF1AX are associated with delayed metastasis and no metastasis, respectively [15]. Our previously published web-accessible tool PRiMeUM (Prediction of Risk of Metastasis in Uveal Melanoma) also incorporates patient and clinical information, including age at diagnosis, gender, tumor location, and tumor size measurements, as well as the copy number status of chromosomes 1, 3, 6, and 8, to predict patient risk for metastasis [3]. Finally, the presence of a specific 12-gene expression pattern in combination with chromosome 8q loss can be used to predict risk of metastasis [16,17,18]. 

These existing clinical and molecular prognostic biomarkers are used to stratify patients based on risk of metastasis to appropriate management and surveillance protocols, as well as to identify those who could benefit from clinical trials for adjuvant therapy aimed at reducing risk of metastasis. However, the type of biomarker(s) used is highly variable between centers, and to date, has not resulted in a reduction in UM mortality as therapeutic vulnerabilities have not been linked to these biomarkers. Further, the accuracy of the models remains imperfect, partially due to the reliance on small and heterogeneous cohorts [15]. Thus far, methylation biomarkers are not routinely used in clinic, and thus represent an additional dimension of information that may increase the accuracy of prognostic models and/or identify novel biology linked to therapeutic vulnerabilities.

Relative to other cancers, UM has a low mutation burden, and it has been proposed that UM may be more influenced by epigenetic drivers of cancer progression and metastasis than by specific DNA variants [19]. While the association of various clinical and genetic features with metastasis in UM have been well studied, the role of epigenetic factors has only recently started to gain attention [20,21]. Hypermethylation of genes such as CDKN2A (16INK4a) [22], RASSF1A [23,24,25], RASEF [26], EFS [27],TERT [28], and BAP1 [14,29] have been studied using individual gene promotor methylation assays and linked to metastasis or poor prognosis. More recent genome-wide methylation studies [30,31,32,33,34,35] have revealed additional genes and biological pathways as potential prognostic biomarkers and therapeutic targets, highlighting the critical role of methylation changes in UM.

The purpose of this retrospective study is to identify DNA methylation patterns in primary UM (pUM) in individuals with known metastatic outcomes, utilizing a new cohort of 41 pUM tumors in addition to 69 pUM tumors from the Cancer Genome Atlas (TCGA) Project [35]. DNA methylation is a stable and long-lasting modification, thus allowing both recently collected and archived samples to be tested reliably. Overall, the aim is to provide an improved classification scheme that will add to the current list of prognostic indicators, thereby enabling a more accurate prediction of metastasis, through the identification of a unique DNA methylation pattern in pUM. These changes in DNA methylation may be the consequence of the genetic aberrations already detectable in pUM, and/or may reveal other genes, genomic regions, or biological pathways that contribute to cancer progression. Furthermore, a multigene methylation signature associated with poor prognosis in pUM could substantially improve the accuracy of models available to physicians to predict an individual’s risk of metastasis at the time of pUM diagnosis.

## 2. Materials and Methods

### 2.1. pUM Samples

The pUM dataset was composed of two independent sets of primary tumor samples. The first, the GDL cohort, was a group of 41 UM individuals diagnosed with pUM and managed by the Ocular Oncology Service at Wills Eye Hospital, Philadelphia, Pennsylvania between 1989 and 2013. Cases included in this study were drawn from those submitted to the Genetic Diagnostic Laboratory (GDL), University of Pennsylvania. Inclusion was based on the availability of complete clinical information, availability of banked DNA, and information on whether metastases had occurred (full details available in Supplemental Table S1). UM samples collected following enucleation (N = 32) or fine-needle aspiration biopsy (N = 9) were submitted to GDL for chromosomal copy number analysis. Information on age at time of diagnosis, sex, and tumor location and size were collected at the time the samples were submitted to GDL. Tumor dimensions were determined at the time of initial diagnosis. Information on UM metastasis and follow-up time was obtained by a retrospective review of medical charts. Informed consent for the use of excess tissue and relevant information for research purposes was obtained from all individuals who submitted samples for chromosomal testing. This research was approved by the Institutional Review Board of the University of Pennsylvania. 

The second set of UM methylation data was extracted from the data for 80 patients with UM analyzed as part of the TCGA project [35]. Data were obtained via the TCGA Data Portal and TCGAbiolinks. Samples with less than 12 months of follow-up time without a documented metastasis were removed from the cohort for all TCGA analyses (*n* = 11). 

The median metastasis-free time was 7.2 years and 2.6 years for the GDL and TCGA cohorts, respectively.

### 2.2. DNA Extraction, Bisulfite Conversion and Array Hybridization

DNA was extracted from pUM fine-needle aspirate biopsy and enucleated tumor samples as previously described [2]. The DNA was bisulfite-converted and hybridized to the Illumina Infinium HumanMethylation 450K BeadChip (Illumina, San Diego, CA, USA) (N = 24), and the Illumina Infinium Methylation EPIC BeadChip (N = 17), following manufacturer’s protocols.

### 2.3. Data Normalization

Methylation data for the GDL cohorts from both 450K and EPIC arrays were merged into one “virtual” 450K array, including 452,567 probes, for joint normalization and downstream analysis. Normalization was performed using the single-sample Noob method (“preprocessNoob” function) as suggested for merged 450K and EPIC arrays [36]. Probes with low detection values (*p*-value < 1%; N = 4788) or with a bead count <3 (N = 250) were removed. Probes with an SNP at the hybridization site, CpG interrogation site, or at the single nucleotide extension site; probes previously annotated as cross-reactive probes [37,38]; and probes on the sex chromosomes were removed. To account for potential underlying batch effects between 450K and EPIC arrays and plates, batch-effect correction was applied for the five plates with the ComBat package [39]. Principal component analysis (PCA) was used to evaluate for potential confounding variables; no batch effect between plates was apparent. Normalized data are available from the GEO database (GSE270084).

For the TCGA cohort, the processed UM methylation data for all 80 samples from the original TCGA study were retrieved from TCGAbiolinks. Probes with “NULL” values were removed and beta values were converted to M-values. The GDL and TCGA datasets were merged to include only probes common to both (N = 286,168 probes and 121 samples), and the merged dataset was batch-corrected according to origin (TCGA vs. GDL) using the ComBat package. Probes with low methylation values in the GDL cohort (beta value < 0.3 in >90% samples) were removed from downstream analyses.

### 2.4. Data Analysis and Visualization

Analyses were performed using R (Version 3.4.4 (15 March 2018)) and with the hg19/GRCh37 human genome reference. After normalization of beta values, M-values were used for data analysis and visualization. Visualization was performed using R packages ComplexHeatmap (v2.16.0) [40], ggplot2 (v 3.4.2) [41] and BoutrosLab.plotting.general (v 7.0.8) [42]. The GDL cohort was used as the discovery cohort, and the TCGA cohort as the validation cohort. For statistical analyses, probe methylation was compared between patients who went on to develop metastasis and those who remained metastasis-free (the reference group).

### 2.5. Differentially Methylated Probes (DMPs) and Univariate Prognosis

The top 25% most variable probes (N = 46,057) based on median absolute deviation (MAD) were used to identify probes on a univariate basis, either based on differential methylation and/or univariate prognosis. DMPs were detected by using the dmpFinder function from the minfi package [43], and also with a nonparametric approach using the Wilcoxon/U-test to assess significance of the median methylation change. Probes with positive and negative methylation changes represent hypermethylated and hypomethylated probes, respectively, in patients who developed metastasis. To assess prognostic impact, a univariate hazard ratio was calculated for each probe with a Cox proportional hazard model, comparing the patients with methylation values in the top tertile for that probe to the remaining patients. A second model adjusting for age and sex, which are known variables to influence methylation, was also computed.

### 2.6. Cluster Analysis

Probe selection for unsupervised analysis was based on univariate prognosis (unadjusted Wald *p*-value < 0.05 and adjusted Wald *p*-value < 0.01) and/or differential methylation (Wilcoxon/U-test adjusted *p*-value < 0.05 OR absolute changes in methylation greater than 3). Only probes with an absolute change in methylation greater than 2 were considered. In total, 622 probes met this criterion in the GDL cohort. The GDL cohort was clustered with ConsensusClusterPlus (v1.64.0) [44] using 1000 iterations, and the Spearman distance and PAM clustering methods with 80% resampling of patients and probes per iteration. The optimal number of clusters was selected based on visual review. TCGA samples were assigned to GDL cluster using the centroid approach. 

### 2.7. MethylSig-UM Signature Development

Probes with an absolute change in methylation greater than 1.0 were used for supervised machine learning (N = 11,402 probes remaining). Highly correlated probes (R-squared > 0.9) were removed (1215 removed, 10,187 remaining). A custom resampling algorithm was designed, with 1000 bootstrap iterations, holding out 5% of samples each time. For each iteration, univariate probe ranking based on univariate prognosis and methylation change was used, with the same rules as described above in the cluster analysis. Recursive feature elimination (RFE, from R package caret v6.0-94) [45]) with 10-fold cross-validation was used to evaluate a range of biomarker sizes (5, 10, 15, 25, 50, 75, 100, 150, 200, 250, and 300 probes). For each iteration, the optimal biomarker size was selected based on accuracy, and selected probes as well as their variable importance were tabulated. RFE works by iteratively removing the least important features from a model until a desired number of features is reached, and aims to improve model performance, reduce overfitting, and enhance interpretability by selecting the most relevant features. The final biomarker size was determined by the median accuracy of the various biomarker sizes, and probes were selected based on the frequency of inclusion in cross-validation models. The code to run MethylSig-UM is freely available on github (https://github.com/emlalonde/MethylSigUM/, accessed on 18 June 2024).

### 2.8. Differentially Expressed Genes

RNA sequencing data were downloaded for the 69 UM analyzed as part of the TCGA project. Raw feature counts on Ensembl protein-coding genes were extracted from each individual data file. DESeq2 (v1.40.2) [46] was then used to detect differentially expressed genes with default parameters.

### 2.9. GO Term Analysis

Over-representation analysis was performed with the WebGestalt program (online version 2024) [47] using GO Biological process terms, nonredundant. Redundancy of the GO terms was removed by the “Weighted set cover” option.

## 3. Results

### 3.1. Patient Demographics

The GDL cohort used in this study included 41 UM primary tumor tissue samples collected from patients at the time of fine-needle biopsy or enucleation at Wills Eye Hospital in Philadelphia, Pennsylvania between 1989 and 2013 (Table 1). The TCGA cohort was used as a validation cohort. Of note, both cohorts lack patients from the American Joint Committee on Cancer (AJCC) T1 group, and thus represent patients with larger and more aggressive tumors. 

### 3.2. Differential Methylation Analysis

**Figure 1:**
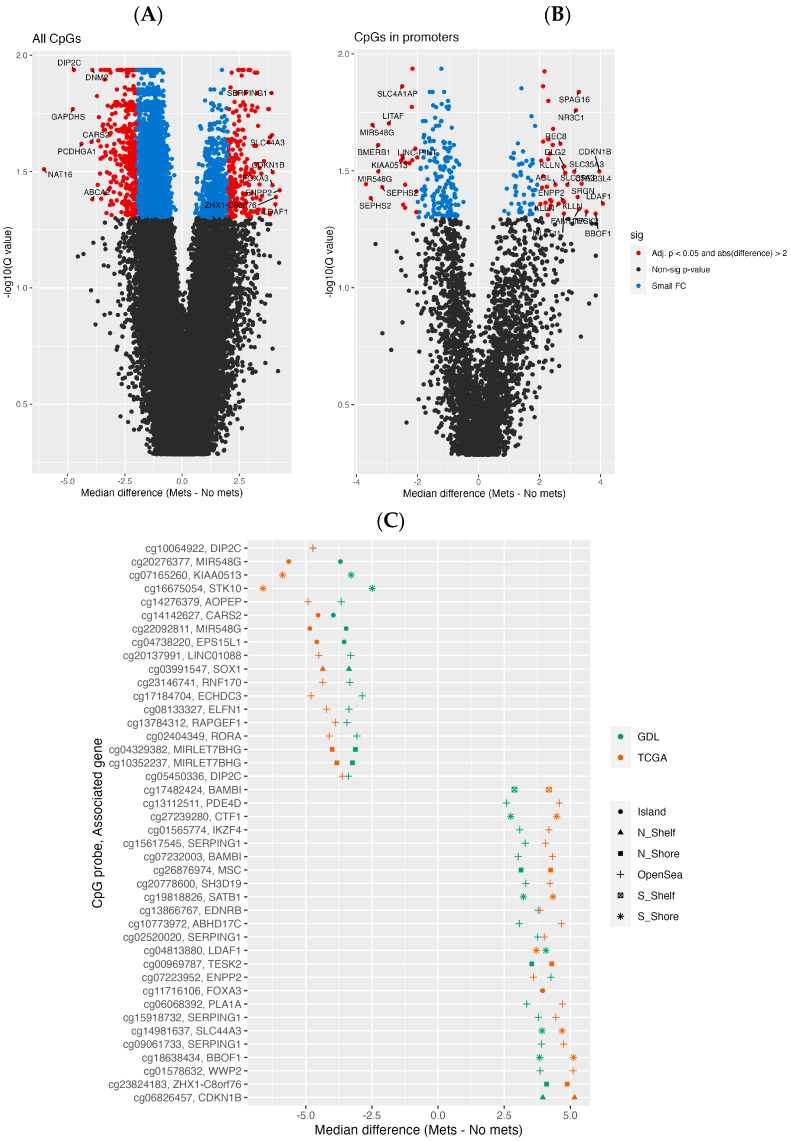
Differential methylation in primary uveal melanoma. Methylation difference from tumors in patients who developed metastasis (“Mets”) compared to those who did not (“No mets”). (**A**) GDL cohort considering all probes; (**B**) GDL cohort considering only probes in promoter regions; (**C**) probes with largest absolute fold changes from both cohorts.

Overall, there was a difference in degree of methylation of CpG probes in patients with pUM from the GDL cohort who developed metastasis, compared to patients who did not develop metastasis, including more hypermethylated probes in gene promoter regions (Figure 1A,B, Supplemental Table S2). Methylation differences between the two cohorts were largely concordant, with more significant probes in the TCGA cohort (Supplemental Figure S1). Using a threshold of absolute change in median methylation greater than 2 and an FDR-adjusted *p*-value < 0.05, there were 604 and 2661 differentially methylated probes (DMPs) in the GDL and TCGA cohorts, respectively, with 267 probes shared between the two cohorts. Of the probes with the largest median difference in both cohorts (Figure 1C), a probe 1.3 kb upstream of the CDKN1B gene and a probe in the open sea of the DIP2C gene region were the CpG sites with the highest and lowest methylation changes, respectively. There were no DMPs in genes known to be involved in UM tumorigenesis.

### 3.3. Epigenomic Subtype Analysis

**Figure 2:**
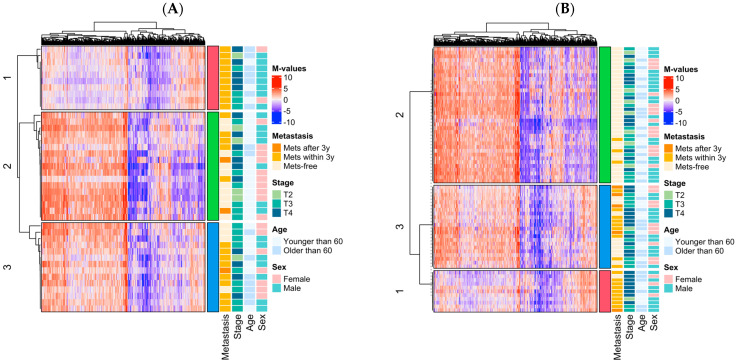

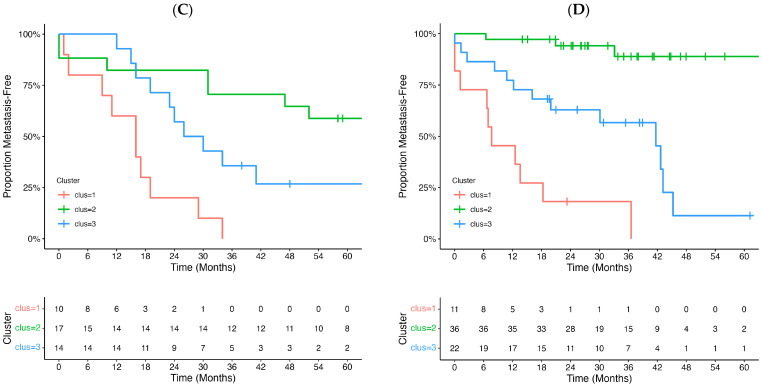
Epigenomic subtypes of primary uveal melanoma. (**A**) Consensus clustering of the GDL cohort (rows) based on 622 probes (columns) defines three epigenomic clusters. (**B**) TCGA cohort mapped to GDL clusters in (**A**). Gene order is based on clustering of GDL cohort, and patients are ordered within each cluster based on Ward clustering using the Spearman distance metric. (**C**) Prognostic assessment of the epigenomic clusters in the GDL cohort. (**D**) Prognostic assessment of the epigenomic clusters in the TCGA cohort.

Unsupervised clustering was undertaken in the GDL cohort to investigate epigenomic subtypes of primary UM. Probes were selected based on differential methylation or prognostic significance, resulting in 622 probes for consensus clustering (Supplemental Table S3). Three clusters were defined in the GDL cohort (Figure 2A) and TCGA samples were assigned to these clusters based on similarity to the median methylation profile of the cluster within the GDL cohort (Figure 2B). In both cohorts, the clusters were associated with metastasis-free survival, with Cluster 1 being associated with the worst prognosis and Cluster 2 with the best prognosis (Figure 2C,D). In a multivariate model adjusting for pertinent clinical variables in each cohort, the cluster grouping remained strongly associated with risk of metastasis (Table 2A,B). Cluster 1 showed hypomethylation in more than half the probes, with one distinct group of hypermethylated probes, including probes previously identified as hypermethylated in gene expression profiling (GEP) Class-2 UM in the ENPP2 and EDNRB genes [14].

Overall, the differential methylation and delineation of prognostic epigenomic subtypes suggests that global DNA methylation is different in patients with pUM who progress to metastasis, and can be used for stratifying patients into different prognostic groups at the time of diagnosis.

### 3.4. Prognostic Signature Development

**Figure 3:**
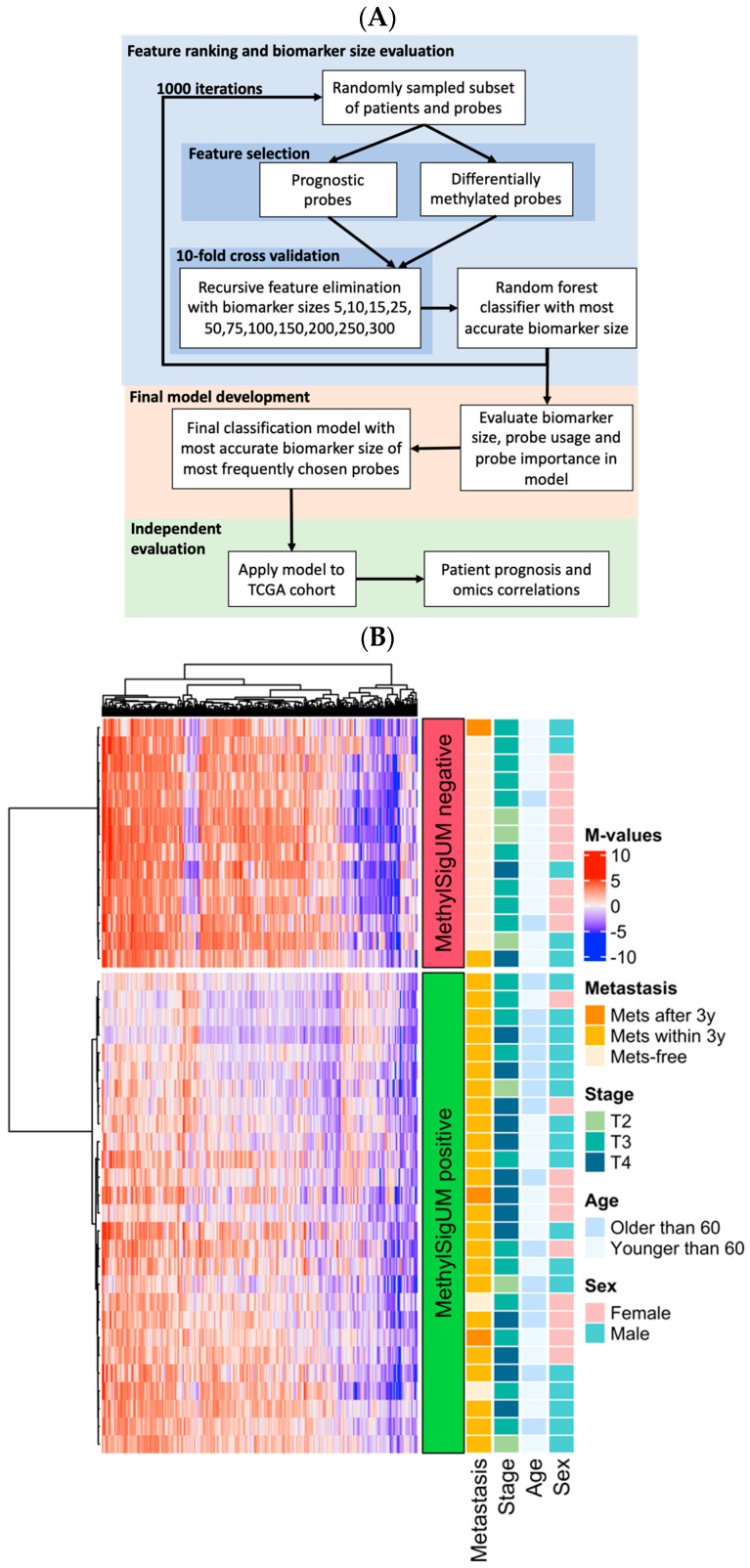
Development of 250 CpG probe epigenomic signature, MethylSig-UM. (**A**) Machine learning framework used to rank and select candidate features, build model, and test model performance. For feature selection, 1000 iterations using random subsets of the data were used to fit models of various sizes via cross-validation. The features used for the best model were recorded. The final biomarker size and the final probes used for the signature are based on the results from the 1000 bootstrap iterations. Model performance was estimated in the TCGA cohort, which was not used for feature selection or model training. (**B**) Heatmap of 250 probes in the GDL cohort, split by predicted class (MethylSig-UM prediction). Rows (patients) are clustered within their predicted classes and genes (columns) are clustered.

To develop a methylation signature to identify newly diagnosed patients with pUM at highest risk of metastasis, a custom supervised machine learning strategy was developed (Figure 3A). Due to the small cohort size with a large feature set, a repeated cross-validation feature selection strategy was employed, following by random forest modeling which is known to reduce the risk overfitting. In brief, feature selection was performed using the GDL cohort with a repeated cross-validation strategy, testing biomarker sizes from 5 to 300 probes via recursive feature elimination, which iteratively removes lowest ranking features. The optimal biomarker size was determined based on the median classification accuracy of the cross-validation models. A random forest model was then trained on the full GDL cohort using this biomarker size with the most commonly selected probes from the repeated cross-validation iterations, and the metastasis status as the prediction class. The TCGA was used as an independent test cohort. 

The final methylation signature, named MethylSig-UM, was composed of the 250 most commonly selected probes (Supplemental Table S4), as this signature size was most commonly selected and was found to be the most accurate in cross-validation (Supplemental Figure S2A,B). Most probes were chosen in fewer than 50/1000 iterations, whereas 137 were selected in at least half of the iterations (Supplemental Figure S2C). In the final model, the frequency of probe selection was weakly correlated with feature importance, as measured by the decrease in Gini impurity index (Pearson correlation R^2^ = 0.363, *p* = 3.97 × 10^−26^; Supplemental Figure S2D). The probe with the highest variable importance, cg23480341, which is upstream of the SLPI gene, was also the most selected probe (941 times), whereas the second most important probe, cg24360137, an intergenic probe, was selected in fewer than half of the iterations (421/1000). Probes in genes of interest in the signature included nine probes from the gene PTPRN2, three from PCDHGA1, two in MGMT, and two each in the kallikrein subgroup of serine proteases KLK2 and KLK5. Most probes (75.6%) in the signature were hypermethylated in good-prognosis patients compared to those who developed metastasis, and overall, the probes selected had either high or low methylation in good-prognosis patients compared to neutral values in patients who developed metastasis (absolute median methylation M-values 2.97 vs. 0.789; Figure 3B). The exception was one small group of probes (N = 20) which were strongly hypomethylated in patients who developed metastasis. One hypomethylated probe is located within the promoter of the TAGLN2 gene, consistent with a previous study showing upregulation of TAGLN2 is associated with invasion, poor prognosis, and metastasis [48]. In several other neoplasms, TAGLN2 promoter hypermethylation is also observed in patients with a good prognosis [49].

**Figure 4:**
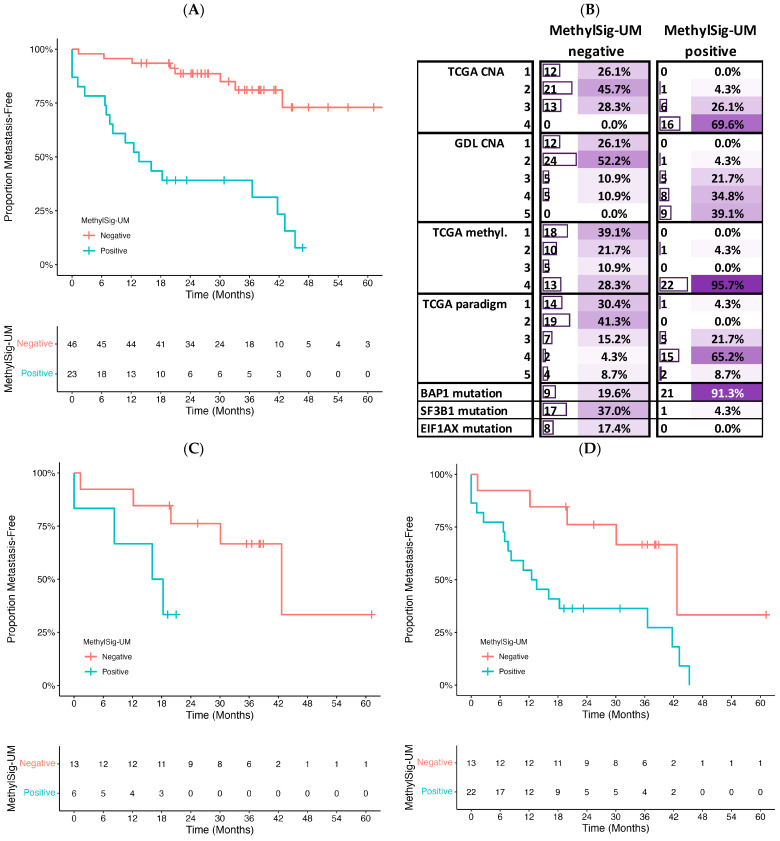
Validation of MethylSig-UM in the TCGA cohort. (**A**) Kaplan–Meier curve of TCGA cohort based on stratification by MethylSig-UM. (**B**) MethylSig-UM classification compared to other established omic subtypes including TCGA CNV subtypes, GDL CNV subtypes [50], TCGA methylation subtypes, and TCGA Paradigm subtypes. The number (bar chart) and percent (heat map) of patients are indicated for each omic biomarker, stratified by MethylSig-UM. The color in the heat map ranges continuously from white for 0% to dark purple for 100%. Within each omic subtype, the percentages refer to percent of patients within MethylSig-UM class positive, as per the original TCGA publication. (**C**) Kaplan–Meier curve of TCGA patients from TCGA CNA cluster 3 further stratified by MethylSig-UM. (**D**) Kaplan–Meier curve of TCGA patients with monosomy 3 further stratified by MethylSig-UM.

When applied to the TCGA cohort as an independent validation, MethylSig-UM was able to distinguish patients at high risk of metastasis with an area under the receiver operator curve (AUROC) of 81% (Figure 4A). Cox proportional hazard modeling revealed that patients positive for MethylSig-UM exhibit a hazard ratio of 7.2 (95% confidence interval 3.08–16.8; *p* < 0.0001), while adjusting for tumor stage (Table 3A). Further, adding MethylSig-UM to a validated clinico-genomic prognostic model, PriMeUM, which accounts for tumor size, patient sex, and age as well as prognostic CNAs, MethylSig-UM remained prognostic while PriMeUM was borderline significant in the validation cohort (Table 3B).

Comparing MethylSig-UM to other published classification schemes showed similar groupings with the signature-positive patients largely mapping to other high-risk groups. For instance, all but one patient positive for MethylSig-UM mapped to the highest risk methylation cluster (cluster 4) from the original TCGA study, despite completely different methodology (Figure 4B). Compared to genomic subgroups based on CNAs, MethylSig-UM-positive patients included all patients from the highest risk TCGA CNA cluster (cluster 4), and MethylSig-UM remained prognostic within the second-highest risk group, cluster 3 (Figure 4C). More specifically, 6/19 patients in TCGA CNA cluster 3 were positive for MethylSig-UM, all of which had at least one high-risk genomic feature, and four of six developed metastasis; the remaining two patients had a follow-up time <24 months. MethylSig-UM also identified all nine patients who met the criteria for the ultra-high-risk CNA cluster, as defined by our group based on the presence of monosomy 3, amplification of 8q, and deletion of 1p or 16q [50]. Critically, in patients with monosomy 3, ~60% of them were positive for MethylSig-UM, and indeed had a 2-year metastasis rate of 63.6% compared to only 23.8% for the MethylSig-UM negative subset (Figure 4D). Finally, compared to the TCGA integrated “PARADIGM” clusters based on mRNA, CNAs, and pathway interactions, MethylSig-UM-positive patients mapped to the three highest risk groups, and only MethylSig-UM and the highest risk integrated cluster (cluster 5) remained significant in a multivariate prognostic model (Table 2C).

**Figure 5:**
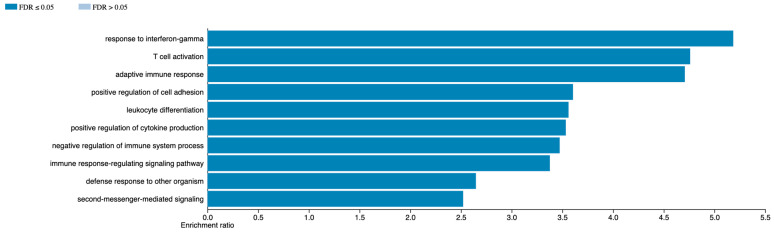
GO pathway over-representation analysis based on differentially expressed genes in MethylSig-UM-positive patients. Figure generated by the WebGestalt program using GO Biological process terms, nonredundant. Redundancy of the GO terms was removed by the “Weighted set cover” option.

To investigate the potential biological mechanisms of MethylSig-UM, differential gene expression analysis was performed between patients positive and negative for the methylation signature using the public TGCA RNA expression data. There were 554 upregulated genes and 549 downregulated genes, with a log fold-change greater than 1.5 and adjusted *p*-value < 0.05. Over-representation analysis of GO biological terms using upregulated genes in the positive–positive patients identified several GO terms related to immune response, including response to interferon gamma (enrichment ratio 5.2), T-cell activation (enrichment ratio 4.8) and adaptive immune response (enrichment ratio 4.7), as the three highest terms (Figure 5). Downregulated genes resulted in only one significant GO term (cell–cell adhesion via plasma membrane adhesion molecules).

## 4. Discussion

Multiple prognostic biomarkers are available to guide pUM patient management based on the predicted risk of recurrence, including CNA biomarkers, RNA expression signatures, and somatic variants [11,12,13,14,15,16,17,18]. Despite these advances in multi-omic prognostic biomarker detection for pUM, clinical outcomes for those with high-risk disease remain poor. There is thus an unmet need to better understand the underlying biology of these patients with the goal of identifying more precise therapeutic interventions.

Aberrant DNA methylation is a common phenomenon in cancer and is associated with epigenetic reprogramming, increased chromosomal instability, and induction or repression of gene expression [51,52]. The molecular consequence of methylation changes can be difficult to predict, particularly for those CpG probes outside of promoter areas, where an inverse association with gene expression could be predicted. For the remaining probes, additional evidence at the mRNA and/or protein level are needed to predict functional consequences, as methylation of a CpG probe may influence distant genes and/or have little or subtle effects on gene expression. Other studies have explored such methylation associations with mRNA abundance [30,33]. In this study, we focused uniquely on changes in methylation associated with metastatic risk, and relied upon the literature to put our findings into context. We utilized the public TCGA mRNA data to investigate dysregulated pathways in patients positive for our novel epigenetic signature, MethylSig-UM.

Specifically, we performed genome-wide methylation analysis of 41 patients with pUM with AJCC stage T2–T4 and with an average of 7.2 years of metastasis-free follow-up time. These data showed a clear difference in the overall DNA methylation patterns between primary tumors that progressed to metastasis and those that did not. A probe 1.3 kb upstream of CDKN1B, a tumor suppressor associated with autosomal dominant multiple endocrine neoplasia type IV (MEN4, OMIM 610755), was the highest ranking differentially methylated probe. Hypermethylation in patients with metastasis may indicate transcriptional silencing of this tumor suppressor, resulting in increased metastatic potential. Indeed, in a TCGA pan-cancer analysis, uveal melanoma had one of the strongest associations between patient outcomes and CDKN1B mRNA expression, with higher expression observed in patients with higher rates of metastasis [53].

Differential methylation was observed at the level of single CpG loci but also unsupervised analysis detected three patient epigenomic subtypes based on these differentially methylated loci. The original TCGA study defined four methylation subtypes although clusters 1–3 have similar methylation and prognostic profiles. While it can be challenging to derive biological meaning from unsupervised clustering results of DNA methylation, we note that one of the most differentially methylated probes in our high-risk subtype, Cluster 1, is located within the LINC01088 noncoding RNA; this probe is hypomethylated in cluster 1 compared to clusters 2 and 3. LINC01088 overexpression has recently been shown to facilitate colorectal cancer progression by regulating key miRNAs resulting in immune escape. Conversely, two probes associated with the ENPP2 gene are hypermethylated in cluster 1 compared to clusters 2 and 3. ENPP2 encodes the autotaxin enzyme which promotes tumor cell migration and metastasis [54]. Hypermethylation with lower *ENPP2* gene expression has previously been reported in UM, including enrichment in gene expression profiling Class-2 (BAP1-mutated) UM [14,34]. Interestingly, in the TCGA study, reduced ENPP2 expression was found to differentiate the highest risk copy number subtype from the second-highest risk subtype; this was inversely correlated to ENPP2 methylation and was associated with poor prognosis [35]. Higher methylation of the ENPP2 promoter region is also associated with poor prognosis in prostate and colon cancers [55].

Finally, to compare the prognostic ability of methylation to other omic biomarkers, a custom supervised machine learning strategy using recursive feature elimination and random forest was applied to generate a methylation-specific prognostic biomarker. This resulted in a 250-probe methylation signature, named MethylSig-UM, which performed very well on the independent validation set, the TCGA cohort (AUROC: 81%). In multivariable Cox proportional hazard models, the signature remained highly prognostic when adjusting for tumor stage (hazard ratio (HR) 7.2, *p*-value 5 × 10^−6^) and a previously validated clinico-genomic model incorporating CNVs, PRiMeUM (HR 4.5, *p*-value 0.0016), as well as the TCGA-developed multi-omic Paradigm clusters (HR 5.6, *p*-value 0.0075).

Further, MethylSig-UM may identify a subset of patients with pUM with a unique underlying biology which are currently distributed among various published omic-subgroups, albeit enriched for the higher-risk subtypes (Figure 3B). For instance, MethylSig-UM-positive tumors are also classified as high-risk based on TCGA copy number subgroups, and the signature remains prognostic within the second-highest TCGA CNA cluster as well as within patients with monosomy 3. All patients classified as “ultra-high-risk” based on our recent CNA classification schema also tested positive for this signature. Finally, 91% of MethylSig-UM-positive patients harbor a BAP1 mutation, also associated with poor prognosis. Thus, MethylSig-UM synergizes with the well-established prognostic biomarkers and shows potential to complement these existing UM biomarkers to further improve the accuracy of patient prognostication, even in those with monosomy 3.

Similar to the high-risk epigenomic cluster defined in this study, the high-risk patients identified by MethylSig-UM showed primarily loss of hypermethylation in the 250-probe signature, with one small group of particularly hypomethylated probes. Thus, the epigenomic profile associated with pUM patients with high metastatic potential tends to be largely characterized by loss of methylation, potentially resulting in spurious activation of gene expression. Altered gene expression associated with these hypomethylated probes may be candidate targets for prevention of metastatic spread. Since the CpG probes may not act directly on the nearest gene, the TCGA mRNA data were used to identify altered pathways in patients positive for MethylSig-UM. This differential expression and GO term enrichment analysis revealed that patients positive for MethylSig-UM had upregulation of genes related to response to interferon gamma, T-cell activation, and adaptive immune response, among others. It is known that interferon gamma (IFNG) can play a significant role in innate and adaptive immune response. It has been proposed that IFNG has an important role in immunoediting, which means reprogramming of cancer cells such that they survive immune-mediated cell death [56]. This would explain why MethylSig-UM-positive cases are associated with poor prognosis. It is also known that unlike cutaneous melanoma, uveal melanoma is refractory to Immune Checkpoint Blockade therapeutics [57]. While there are many speculations as to why this is, it is for certain that pUM has a lower tumor mutation burden, and hence lower neo-antigen presentation, resulting in lower T-cell-mediated cancer cell clearance. Therefore, MethylSig-UM may identify, at the time of diagnosis, pUM cases which will not respond to standard immunotherapeutic approaches. Instead such treatment can lead to even poorer prognosis [58,59]. Together, these findings indicate that DNA methylation is predictive for metastatic risk along with age at diagnosis, sex, tumor location and size measurements, and other omic biomarkers, and suggests that MethylSig-UM may identify patients with immune dysregulation refractory to immunotherapeutics.

Methylation classifiers are being increasingly used in molecular pathology diagnostics to provide more precise diagnoses (e.g., in brain tumors [60], bone and soft-tissue sarcomas [61], B-cell lymphomas [62], and others), to identify signatures associated with genetic mutations [63,64], as well as to evaluate biological processes. In UM, a biomarker developed from a methylation array could be used to score patients for methylation signatures, but also to assess for copy number status, as these arrays can measure both methylation and CNVs. Further, building upon recent work detailing differentially methylated regions in UM patients with BAP1, SF3B1, and EIF1AX mutations [14,29,34], if robust signatures can be developed to identify patients with BAP1, SF3B1, or EIF1AX mutations, methylation profiling could also be used as a proxy for sequencing studies. As tissue availability is a common issue in UM due to most samples being obtained via fine-needle aspirate biopsy, methylation profiling to assess these multi-omic abnormalities is a promising clinical assay. In current clinical practice, if limited DNA is obtained from a UM tumor, oncologists must choose between next-generation sequencing panels, copy number arrays, and/or RNA expression analysis.

Compared to previous studies, the major strength of this study is utilization of an independent cohort with long follow-up time, in addition to the TCGA cohort. Previous studies have largely relied on genomic markers to define high-risk patients, rather than known survival and/or metastatic outcome [14,30], or have used a single cohort without independent validation of results [33,35]. Thus, this dataset is also a rich resource available to enable additional discoveries and validations. The results from this study should be validated in additional independent cohorts. The main limitation of this study is the small sample size of both training and validation cohorts. In addition, not all genetic information was known for patients in the GDL cohort, such as DNA sequence variant status. These are general limitations of studies evaluating UM metastasis risk, due to the paucity of samples, reliance on older samples to enable sufficient follow-up time, and limited DNA available from FNAB specimens. Importantly, these cohorts were biased towards more advanced stages with no AJCC T1 tumors. Given these limitations, it is likely that some of the identified CpG probes may not be generalizable to other pUM cohorts. However, selecting a larger signature size as we did, dilutes the impact of such features, and the validation in the independent TCGA cohort supports the generalizability of the MethylSig-UM signature, as well the correlation of the MethylSig-UM-positive patients with other established high-risk omic biomarkers. Nonetheless, additional studies will be needed to evaluate whether the methylation signature, subtypes, and differentially methylated regions are generalizable to T1 UM patients and to larger, unbiased cohorts. The MethylSig-UM classifier is freely available to enable such efforts.

## Figures and Tables

**Table 1:** Summary of clinical information for GDL and TCGA cohorts.

		GDL	TCGA
Cohort size		41	69 ^1^
Sex	Male	22	41
	Female	19	28
Age at diagnosis	Median	57	60
	Range	(19–77)	(22–86)
AJCC tumor stage	T2	6	10
	T3	20	27
	T4	15	32
Metastasis	No	14	43
	Yes	27	26
Average follow-up time (time to metastasis)	Years	7.2	2.6

^1^ Eleven TCGA samples had metastasis-free follow-up time less than 12 months and were excluded from this study. AJCC: American Joint Committee on Cancer.

**Table 2:** Cox proportional hazard model of the methylation clusters in the GDL cohort. (**A**) Multivariate model in the GDL cohort including the methylation clusters, tumor stage, and patient age at diagnosis; (**B**) multivariate model in the TCGA cohort including the methylation clusters and tumor stage. Note, patient age was not univariately significant in this cohort, so was not included. HR: hazard ratio; CI: confidence interval.

(**A**)			
	**HR**	**95% CI (Low, High)**	***p*-Value**
**Cluster 3 (vs. 2)**	2.14	0.73, 6.27	0.165
**Cluster 1 (vs. 2)**	4.57	1.38, 15.2	0.013
**T4 vs. T2–T3**	3.39	1.44, 7.97	0.0051
**Age (≥60 vs. <60 years)**	2.11	0.87, 5.11	0.097
		**Model Wald *p*-value**	0.00022
		**Model logrank *p*-value**	8.0 × 10^−5^
(**B**)			
	**HR**	**95% CI (Low, High)**	***p*-Value**
**Cluster 3 (vs. 2)**	9.90	2.79, 35.2	0.000405
**Cluster 1 (vs. 2)**	31.6	8.00, 12.4	8.2 × 10^−7^
**T4 vs. T2–T3**	1.92	0.82, 4.50	0.136
		**Model Wald *p*-value**	5.0 × 10^−6^
		**Model logrank *p*-value**	2.0 × 10^−10^

**Table 3:** Cox proportional hazard model of MethylSig-UM in the TCGA cohort. (**A**) Multivariate model including MethylSig-UM and tumor stage. Note, patient age was not significant in this cohort, so was not included in the multivariate model. (**B**) Multivariate model including MethylSig-UM and PRiMeUM, a validated prognostic model integrating CNVs with clinico-pathological variables.

(**A**)			
	**HR**	**95% CI (Low, High)**	***p*-Value**
**MethylSig-UM**	7.20	3.08, 16.8	5.0 × 10^−6^
**T4 vs. T2–T3**	2.35	1.04, 5.33	0.0408
		**Model Wald *p*-value**	7.0 × 10^−6^
		**Model logrank *p*-value**	2.0 × 10^−7^
(**B**)			
	**HR**	**95% CI (Low, High)**	***p*-Value**
**MethylSig-UM**	4.51	1.77, 11.5	0.00162
**PRiMeUM**	2.56	0.9, 17.2	0.0747
		**Model Wald *p*-value**	0.00001
		**Model logrank *p*-value**	3.0 × 10^−7^

## Data Availability

Methylation data for the GDL cohort are available from the NCBI GEO website, GSE270084 (https://www.ncbi.nlm.nih.gov/geo/query/acc.cgi?acc=GSE270084).

## Supplementary Materials

The following supporting information can be downloaded at: https://www.mdpi.com/article/10.3390/cancers16152650/s1, Figure S1: Methylation difference from tumors in patients from TCGA cohort; Figure S2: Feature selection during signature development; Table S1: Detailed clinical information for GDL cohort; Table S2: Differentially methylated probes in the GDL cohort; Table S3: Details of 622 probes used in the clustering analysis; Table S4: Details of 250 probes comprising the MethylSig-UM signature.
