# Supplementary material for: Genome-Wide Methylation Patterns in Primary Uveal Melanoma: Development of MethylSig-UM, an Epigenomic Prognostic Signature to Improve Patient Stratification
Source: Cancers (Basel). 2024 Jul 25;16(15):2650. doi: 10.3390/cancers16152650 (PMC11312132; doi:10.3390/cancers16152650)
Supplement: Supplementary file 1 [file cancers-16-02650-s001.zip › Supplementary Figures S1 and S2 .pdf]

Supplemental Figures

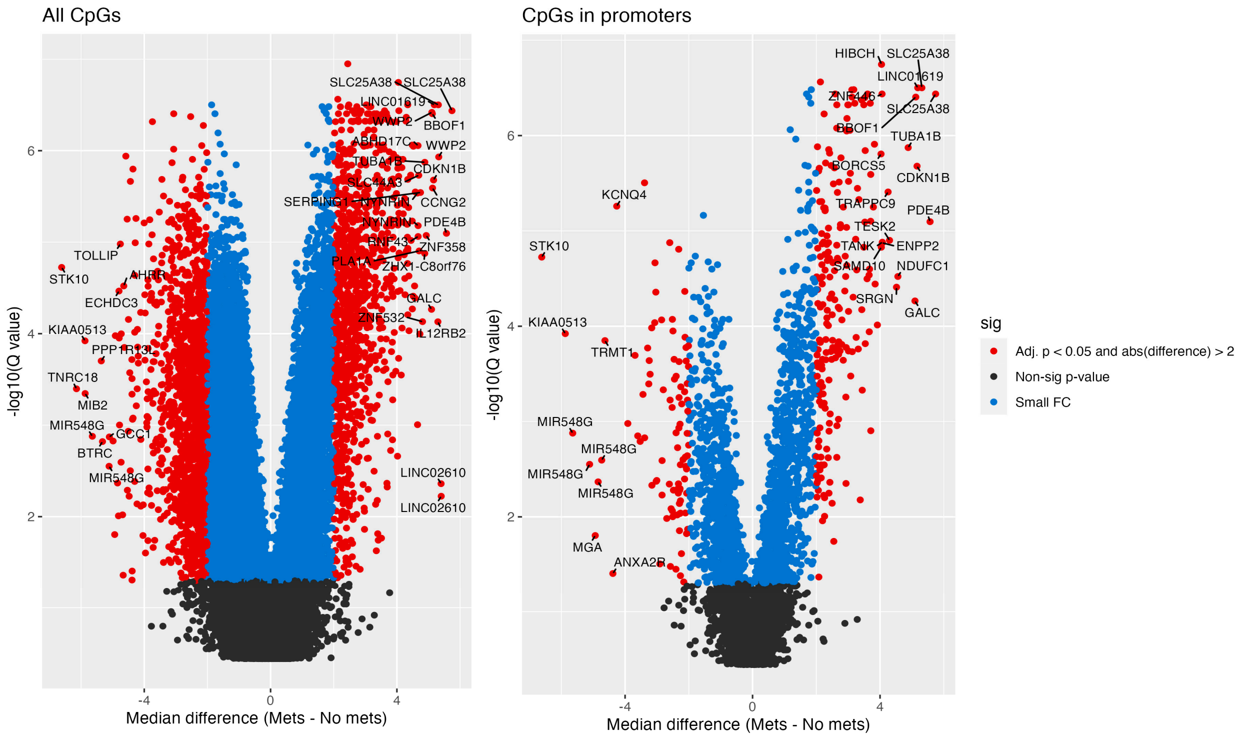

Supplemental Figure 1: Methylation difference from tumors in patients from TCGA cohort who developed metastasis (“Mets”) compared to those that did not (“No mets”). Left: considering all probes; Right: considering only probes in promoter regions

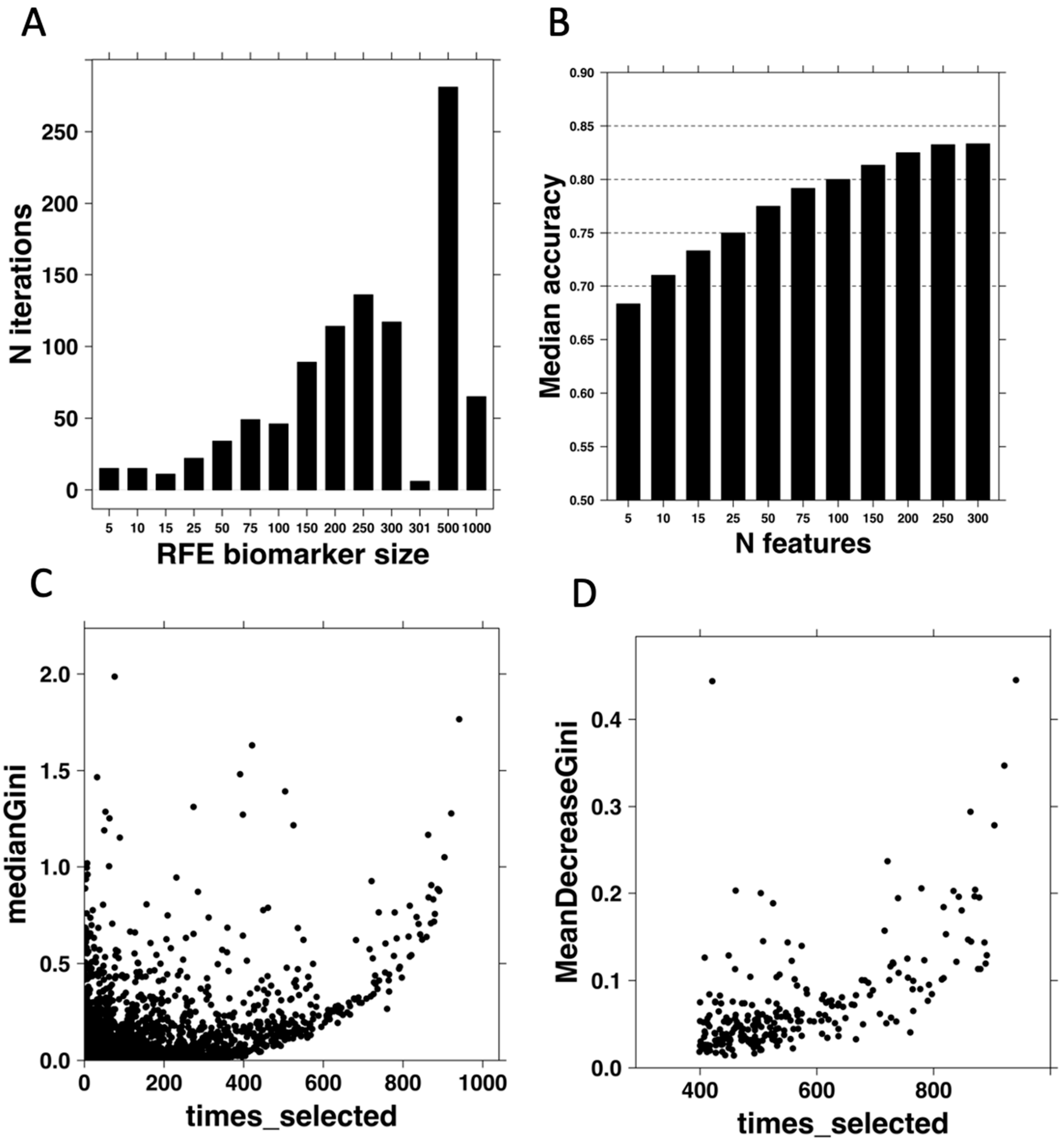

Supplemental Figure 2. Feature selection during signature development.

A) Selected biomarker size per iteration by RFE algorithm

B) Biomarker size vs median classification accuracy

C) Frequency of probe selection compared to median probe importance (gini score from random forest) within the 1000 models used during cross-validation/feature selection. Higher gini scores correspond to higher variable importance.

D) For the 250 probes selected into MethylSig-UM, the frequency of probe selection during cross-validation/feature selection compared to probe importance in the final MethylSig-UM model (gini score from random forest). Higher gini scores correspond to higher variable importance.

Supplemental Tables

Supplemental Table 1. Detailed clinical information for GDL cohort.
Supplemental Table 2. Differentially methylated probes in the GDL cohort.
Supplemental Table 3. Details of 622 probes used in the clustering analysis.
Supplemental Table 4. Details of 250 probes composing the MethylSig-UM signature.
